# ICare.IT: A Longitudinal Study Using Wearable Sensors to Monitor Informal Caregivers of People with Dementia

**DOI:** 10.1093/geroni/igaf122.4261

**Published:** 2025-12-31

**Authors:** Francesca Zanasi, Angela Berardi, Paola Di Florio, Ylenia Druda, Marcello Sicbaldi, Marco Albertini, Marco Domenicali, Alessandro Silvani

**Affiliations:** University of Bologna, Bologna, Emilia-Romagna, Italy; University of Bologna, Bologna, Emilia-Romagna, Italy; University of Bologna, Bologna, Emilia-Romagna, Italy; University of Bologna, Bologna, Emilia-Romagna, Italy; University of Bologna, Bologna, Emilia-Romagna, Italy; University of Bologna, Bologna, Emilia-Romagna, Italy; University of Bologna, Bologna, Emilia-Romagna, Italy; University of Bologna, Bologna, Emilia-Romagna, Italy

## Abstract

Informal caregivers play a vital yet often invisible role in supporting individuals with dementia, facing significant psychological and physical burdens. The ICare.IT study is a novel longitudinal investigation that combines subjective assessments (questionnaires) and objective physiological data (wearable sensors) to evaluate caregiver well-being over time. Critically, this is one of the first studies to apply multiple wearable sensor technology—tracking heart rate, sleep, and physical activity—to a large number of informal caregivers in their daily environment. Participants complete validated online questionnaires and wear sensors for one week, with data collected at baseline (T0), six months (T1), and twelve months (T2). This abstract presents preliminary findings from the first 68 caregivers enrolled in Ravenna (Italy). Most were adult children or partners, with high levels of care intensity (median 42.5 hours/week) and moderate-to-severe burden scores (ZBI median = 39). Notably, 84% reported low social support, and 49% experienced reduced friendship interactions. Anxiety and depression scores were in the borderline range and strongly correlated with perceived burden. Poor subjective sleep quality was also prevalent and associated with increased anxiety, depression, and perceived burden of care. However, heart rate and objective sleep duration were not significantly associated with burden. These early results highlight how wearable technology can reveal new dimensions of caregiver stress, opening pathways for targeted, data-informed interventions. Ongoing follow-up will enrich this analysis and improve understanding of caregivers’ evolving needs.

